# Choroid plexus enlargement in amyotrophic lateral sclerosis patients and its correlation with clinical disability and blood-CSF barrier permeability

**DOI:** 10.1186/s12987-024-00536-6

**Published:** 2024-04-17

**Authors:** Tingjun Dai, Jianwei Lou, Deyuan Kong, Jinyu Li, Qingguo Ren, Yujing Chen, Sujuan Sun, Yan Yun, Xiaohan Sun, Yiru Yang, Kai Shao, Wei Li, Yuying Zhao, Xiangshui Meng, Chuanzhu Yan, Pengfei Lin, Shuangwu Liu

**Affiliations:** 1 Research Institute of Neuromuscular and Neurodegenerative Disease, Department of Neurology, Qilu Hospital, Shandong University, West Wenhua Street No.107, 250012 Jinan, China; 2 School of Clinical Medicine, Shandong Second Medical University, Weifang, China; 3 Department of Neurology, Xiamen Branch, Zhongshan Hospital, Fudan University, 361015 Xiamen, China; 4 Department of Radiology, Cheeloo College of Medicine, Qilu Hospital (Qingdao), Shandong University, Qingdao, China; 5 Department of Radiology, Cheeloo College of Medicine, Qilu Hospital, Shandong University, Jinan, China; 6 School of Nursing and Rehabilitation, Cheeloo College of Medicine, Shandong University, Jinan, China; 7 Department of Clinical Laboratory, Qilu Hospital (Qingdao), Cheeloo College of Medicine, Shandong University, Qingdao, China

**Keywords:** ALS, Choroid plexus, MRI, Blood-CSF barrier

## Abstract

**Background:**

Using in vivo neuroimaging techniques, growing evidence has demonstrated that the choroid plexus (CP) volume is enlarged in patients with several neurodegenerative diseases, including Alzheimer’s disease and Parkinson’s disease. However, although animal and postmortem findings suggest that CP abnormalities are likely important pathological mechanisms underlying amyotrophic lateral sclerosis (ALS), the third most common neurodegenerative disease, no available study has been conducted to thoroughly assess CP abnormalities and their clinical relevance in vivo in ALS patients to date. Thus, we aimed to determine whether in vivo CP enlargement may occur in ALS patients. We also aimed to identify the relationships of CP volume with clinical disabilities and blood-CSF barrier (BCSFB) permeability in ALS patients.

**Methods:**

In this retrospective study, based on structural MRI data, CP volume was assessed using a Gaussian mixture model and underwent further manual correction in 155 ALS patients and 105 age- and sex-matched HCs from October 2021 to April 2023. The ALS Functional Rating Scale-Revised (ALSFRS-R) was used to assess clinical disability. The CSF/serum albumin quotient (Qalb) was used to assess BCSFB permeability. Moreover, all the ALS patients completed genetic testing, and according to genetic testing, the ALS patients were further divided into genetic ALS subgroup and sporadic ALS subgroup.

**Results:**

We found that compared with HCs, ALS patients had a significantly higher CP volume (*p* < 0.001). Moreover, compared with HCs, CP volume was significantly increased in both ALS patients with and without known genetic mutations after family-wise error correction (*p* = 0.006 and *p* < 0.001, respectively), while there were no significant differences between the two ALS groups. Furthermore, the CP volume was significantly correlated with the ALSFRS-r score (*r* = -0.226; *p* = 0.005) and the Qalb (*r* = 0.479; *p* < 0.001) in ALS patients.

**Conclusion:**

Our study first demonstrates CP enlargement in vivo in ALS patients, and continues to suggest an important pathogenetic role for CP abnormalities in ALS. Moreover, assessing CP volume is likely a noninvasive and easy-to-implement approach for screening BCSFB dysfunction in ALS patients.

**Supplementary Information:**

The online version contains supplementary material available at 10.1186/s12987-024-00536-6.

## Background

Amyotrophic lateral sclerosis (ALS) is a rare neurodegenerative disease with both clinical and hereditary heterogeneity [1]. The aetiology of ALS remains unknown; however, interactions between genetic and environmental factors are likely to underpin disease susceptibility [1]. ALS likely derives from cortical influences, and the onset of ALS seems to involve a multistep process with a long preclinical stage [2–5]. Nearly 5–10% of ALS patients carry genetic mutations, most commonly in chromosome 9 open reading frame 72 (C9orf72), superoxide dismutase 1 (SOD1), fused in sarcoma (FUS), and TAR DNA-binding protein 43 (TARDBP), which are responsible for distinct pathological phenotypes [6, 7].

The choroid plexus (CP) is an important epithelial–endothelial vascular structure that resides in the ventricular system and is crucial for maintaining the microenvironment of the central nervous system (CNS) [8–10]. CP epithelia can generate cerebrospinal fluid (CSF), secrete crucial proteins, mediate neuroimmune interactions, and form a blood-CSF barrier (BCSFB) that prevents peripheral blood toxicants from entering the CNS [8–10]. Moreover, the CP is also important for the glymphatic system and may have direct and indirect roles in brain-wide waste clearance [11, 12].

Recently, using in vivo neuroimaging techniques, it has become increasingly apparent that the CP volume is enlarged in multiple neurodegenerative diseases, including Alzheimer’s disease (AD), Parkinson’s disease (PD), frontotemporal dementia (FTD) and multiple sclerosis (MS) [13–18]. For example, Choi et al. reported that a larger CP volume was significantly associated with cognitive deficits across the AD spectrum, and they suggested that CP volume can be used as a neuroimaging marker for clinical prognosis and staging in AD patients [13]. Recently, Assogna et al. found that the CP is enlarged in a large cohort of FTD patients, and they suggested that CP volumetric analysis could represent an imaging marker across the FTD spectrum, particularly at the early stage of disease [16]. Thus, early CP enlargement is likely a common feature and may play an important pathogenetic role in patients with neurodegenerative diseases [13–18].

Against this background, an interesting issue is whether early CP enlargement may also emerge in vivo in patients with ALS, the third most common neurodegenerative disease [1, 19]. Importantly, previous animal and postmortem findings imply that CP abnormalities may exist in ALS patients, either in ALS patients carrying known genetic mutations (defined as genetic ALS patients in this study) or in those without known genetic mutations (defined as sporadic ALS patients in this study), and underpin the pathophysiological process of ALS [20–22]. Moreover, recently, using the CSF/serum albumin quotient (Qalb), a marker of BCSFB permeability, we found that BCSFB integrity is also impaired in ALS [28]. Thus, these studies suggest that, similar to other neurodegenerative diseases, CP enlargement may also occur in ALS. However, to our knowledge, no available study has thoroughly assessed in vivo CP morphology and its correlation with clinical disability and BCSFB permeability in patients with ALS to date, particularly in genetic ALS patients.

Thus, in this retrospective study, we included ALS patients who underwent clinical assessment, genetic testing and structural MRI from a large newly diagnosed cohort of ALS patients. We have three aims. First, we aimed to explore whether CP enlargement can be identified in vivo in ALS patients compared with healthy controls (HCs). Second, according to genetic testing, we divided ALS patients into genetic ALS and sporadic ALS patients, and the CP volumes were further compared in the two ALS subgroups and HCs. Finally, we aimed to explore the relationships between CP volume and clinical disability in ALS patients. Moreover, because calculating the Qalb is an invasive approach to assess BCSFB integrity, and the noninvasive and easy-to-implement nature of assessing CP volume, we further measured the relationships between CP volume and Qalb in ALS patients [13–18, 28].

## Methods

### Participants

In this retrospective study, the inclusion criteria for ALS patients were as follows: (1) newly diagnosed. (2) met the Awaji criteria for probable or definite ALS [25]. (3) underwent clinical assessment, genetic testing and structural MRI scan.

The exclusion criteria for ALS patients were as follows: (1) refusal to participate; (2) inability to complete an MRI scan; (3) combined with FTD, which we chose to exclude because FTD is uncommon (4.7%) in Chinese patients with sporadic ALS [16, 27]. The Rascovsky criteria were used to diagnose FTD [26]; and (4) comorbidity of other neuropsychiatric or systematic disorders.

**Fig. 1:**
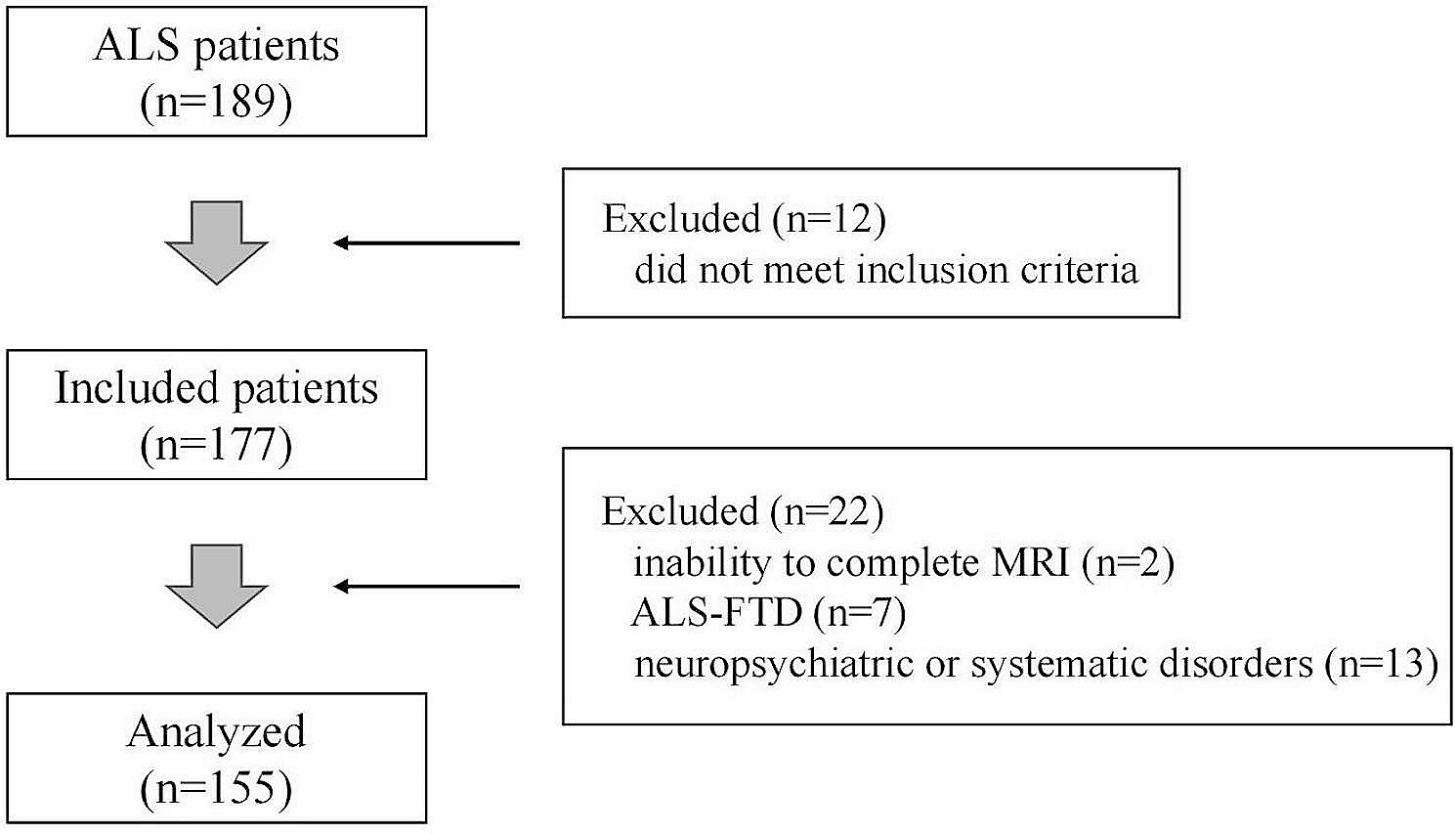
Flow diagram of the inclusion process

Finally, from October 2021 to April 2023, 155 ALS patients were included in this study. In addition, 105 age- and sex-matched healthy controls (HCs) were recruited from the community and were subjected to the same exclusion criteria as the ALS patients. The flow diagram of the inclusion process is shown in Fig. 1.

### Clinical assessments

ALS patients’ demographic and clinical information was recorded, including age, sex, education, family history of neurological diseases, comorbid conditions, site of symptom onset, and disease duration (time from disease onset to diagnosis) [27]. Disease severity was assessed with the Amyotrophic Lateral Sclerosis Functional Rating Scale-Revised (ALSFRS-R) [27]. Baseline progression rate (BPR) was also calculated. Similar to previous studies, BPR was defined as (48 - ALSFRS-R scores)/disease duration [28]. Depression and anxiety were assessed using the Hamilton Depression Rating Scale (HDRS) and Hamilton Anxiety Rating Scale (HARS), respectively [27]. All measures were performed within 2 days of MRI scans, and the clinical information was further corroborated by the primary caregivers.

### Genetic testing

In the present study, similar to our previous study, 32 ALS genes were also screened using whole exome sequencing (WES) in ALS patients (see Supplemental material) [27].

According to genetic testing, a total of 12 mutation carriers were detected, and the detected mutations had an MAF < 0.5% in the population databases (Supplemental Table 1). Thus, these 12 patients were further divided into the genetic ALS group, and the remaining 143 patients were divided into the sporadic ALS group.

### MRI acquisition

All MRI data were obtained on a 3.0 T magnetic resonance system (Philips Medical System Ingenia scanner) with a dStream head coil (8 channel). During the scan, all subjects were asked to be quiet and remain supine. Structural images of the whole brain were scanned using a three‑dimensional (3D) fast spoiled gradient-echo sequence: repetition time (TR) = 6.7 ms, echo time (TE) = 3.0 ms, matrix = 240 × 240 × 170, voxel size = 1 mm×1 mm×1 mm, field of view (FOV) = 240 mm × 240 mm, and a total of 180 slices. FLAIR data were scanned using 2D acceleration, TR = 7000 ms, Flip Angle 90°, TE = 125 ms, acquisition matrix = 272 × 176, and slice thickness 6 mm. The scanning time for all sequences were approximately 11 min.

### CP volume

**Fig. 2:**
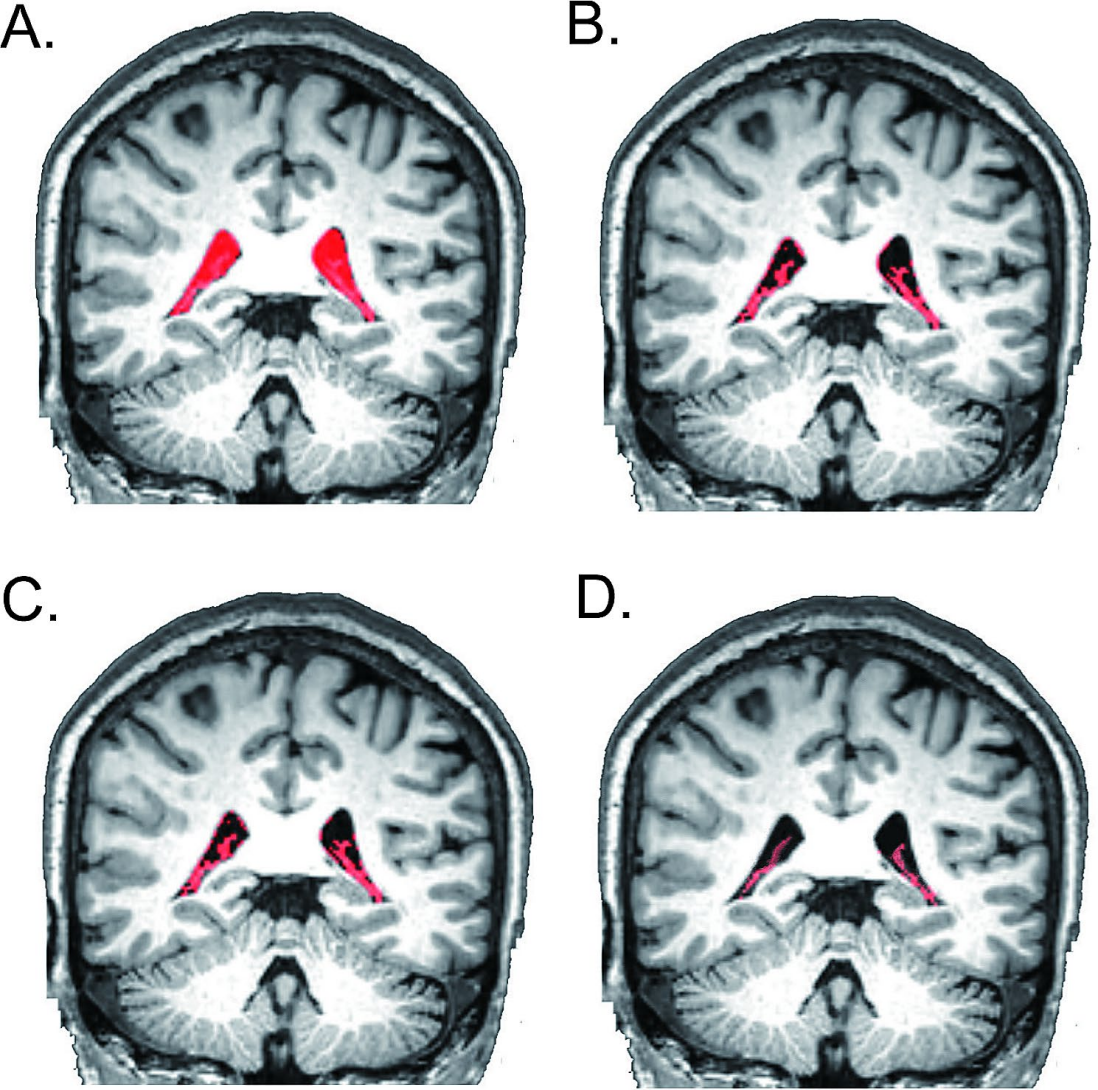
CP segmentation pipeline. (**A**) Using FreeSurfer, the lateral ventricle, inferior lateral ventricle, and CP regions in both the left and right cerebral hemispheres were extracted as the initial mask. (**B**) Bayesian Gaussian mixture models (GMMs) were used to cluster the initial mask into two groups: CSF voxels (low average intensity) and CP and lateral ventricle wall voxels (high average intensity. (**C**) SUSAN smoothing in FSL software was performed on the CP and lateral ventricle wall voxels. (**D**) GMMs were again used to cluster the smoothed voxels into three groups, and the group with the highest average intensity was considered the CP group. Each subject’s CP segmentation was further reviewed and manually corrected by two neuroradiologists, and the CP volume was extracted for further analysis

Similar to previous studies, the CP volume of the lateral ventricles was calculated in this study [15, 18]. First, T1-weighted MR images were auto-segmented into brain cortex regions, WM, and CSF using FreeSurfer software v7.1.1 (http://surfer.nmr.mgh.harvard.edu) [27]. Based on the ‘aparc + aseg.mgz’ file, the lateral ventricle, inferior lateral ventricle, and CP regions in both the left and right cerebral hemispheres were extracted as the initial mask [15, 18]. After that, Bayesian Gaussian mixture models (GMMs) were used to cluster the initial mask into two groups using the scikit-learn page in Python software (https://www.python.org/): CSF voxels (low average intensity) and CP and lateral ventricle wall voxels (high average intensity) [18]. Next, SUSAN smoothing in FSL software (https://fsl.fmrib.ox.ac.uk/fsl/fslwiki/) was performed on the CP and lateral ventricle wall voxels (σ = 1 mm). Then, GMMs were again used to cluster the smoothed voxels into three groups, and the group with the highest average intensity was considered the CP group [18]. Each subject’s CP segmentation was further reviewed and manually corrected by two neuroradiologists (QGR and XSM) who had at least ten years of experience and were blinded to the clinical information and other neuroimaging data. After manual correction, the CP volume was extracted for further analysis. Moreover, similar to our previous study, using FreeSurfer, the total intracranial volume (TIV) was calculated as a covariate [27]. The flow diagram of the CP volume segmentation process is shown in Fig. 2.

### Qalb analysis

In this retrospective study, 115 patients with ALS had available CSF and serum albumin data, and the procedure of lumbar and vein punctures has been described in detail in our previous studies [28]. Then, quotients of albumin were calculated by comparing the level of albumin between serum and CSF (Qalb value = CSF-Alb/serum-Alb) [28].

### Ethics approval

This study was approved by the Research Ethics Committee of the School of Medicine, Shandong University. Participant information was collected only after all patients and HCs had been made aware of the purpose of the study and provided their written informed consent.

### Statistical analysis

For clinical data, continuous variables are reported as the means and standard deviations, and categorical variables are reported as frequencies and proportions. Student’s *t*-tests or analysis of variance (ANOVA) were used to compare continuous variables (with Mann–Whitney *U* tests if necessary). Categorical variables were compared using chi-squared tests. Values of *p* < 0.05 indicated significance. Clinical data analyses were performed using SPSS version 20.0 (IBM Corp., Armonk, NY).

Moreover, general linear models were used to compare CP volume across groups, and age, sex, and total intracranial volume (TIV) were used as covariates. For ALS subgroups and HCs, post hoc tests and family-wise error (FWE) correction were performed, and the threshold for statistical significance was set at *p* < 0.05. Then, partial correlation analyses were performed to identify the relationship of CP volume with clinical data in ALS patients using age, sex, and TIV as covariates. Values of *p* < 0.05 were considered to indicate significant differences.

## Results

### Demographic and clinical information

In the present study, there were no significant differences in age, sex, or education between ALS patients and HCs. Compared with HCs, HDRS and HARS scores were significantly higher in ALS patients (*p* < 0.05). Demographic and clinical information for the ALS patients and HCs is shown in Table 1.

**Table 1:** Demographic and clinical features for ALS patients and HCs

	ALS patients (*n* = 155)	HCs (*n* = 105)	*P*-value
Age (years)	57.1 ± 9.6	57.3 ± 7.9	0.88
Men/Women (n)	94/61	55/50	0.21
Education	9.4 ± 4.0	9.9 ± 3.2	0.26
ALS duration (month)	13.6 ± 7.9	-	-
Bulbar ALS onset n, (%)	39 (25.1)	-	-
ALSFRS-R scores	40.3 ± 3.7	-	-
Progression rate	0.78 ± 0.68	-	-
CP volume (mm^3^)	2930.7 ± 724.8	2454.5 ± 781.3	<0.01
Qalb (mg/g)	5.9 ± 2.8	-	-
HARS scores	8.7 ± 5.2	4.0 ± 3.9	<0.01
HDRS scores	10.5 ± 4.9	3.4 ± 4.2	<0.01

Abbreviations: ALS = amyotrophic lateral sclerosis; HC = healthy control; ALFRS-R = ALS Functional Rating Scale-Revised; HARS = Hamilton Anxiety Rating Scale; HDRS = Hamilton Depression Rating Scale

Moreover, compared with sporadic ALS patients and HCs, age was significantly lower in genetic ALS patients (*p* < 0.05). Compared with sporadic ALS patients, ALSFRS-r scores were lower in genetic ALS patients (*p* < 0.05). Compared with HCs, HDRS and HARS scores were significantly higher in the two ALS groups (*p* < 0.05). There were no significant differences in other clinical data between the two ALS groups and the HCs. Demographic and clinical information for the two ALS groups and HCs are shown in Table 2.

**Table 2:** Demographic and clinical information for patient subgroups and HCs

	Genetic ALS patients (*n* = 12)	Sporadic ALS patients (*n* = 143)	HCs (*n* = 105)	F or χ^2^	*P*-value
Age (years)	44.5 ± 10.3	58.2 ± 8.7	57.3 ± 7.9	14.17	<0.01
Men/Women (n)	9/3	85/58	55/50	2.84	0.24
Education	8.3 ± 3.3	9.5 ± 4.0	9.9 ± 3.2	1.23	0.29
ALS duration (month)	14.5 ± 9.7	13.6 ± 6.0	-	0.37	0.71
Bulbar ALS onset n, (%)	4 (33.3)	34 (23.7)	-	0.54	0.49
ALSFRS-R scores	38.1 ± 6.1	40.5 ± 3.4	-	2.16	0.03
Progression rate	0.75 ± 0.62	0.78 ± 0.69	-	0.13	0.89
CP volume (mm^3^)	3161.8 ± 735.1	2911.3 ± 735.1	2454.5 ± 781.3	13.3	<0.01
HARS scores	8.7 ± 5.8	8.7 ± 5.1	4.0 ± 3.9	30.55	<0.01
HDRS scores	10.1 ± 5.9	10.5 ± 4.9	3.4 ± 4.2	66.92	<0.01

Abbreviations: ALS = amyotrophic lateral sclerosis; HC = healthy control; ALFRS-R = ALS Functional Rating Scale-Revised; HARS = Hamilton Anxiety Rating Scale; HDRS = Hamilton Depression Rating Scale

### CP volume in ALS patients

**Fig. 3:**
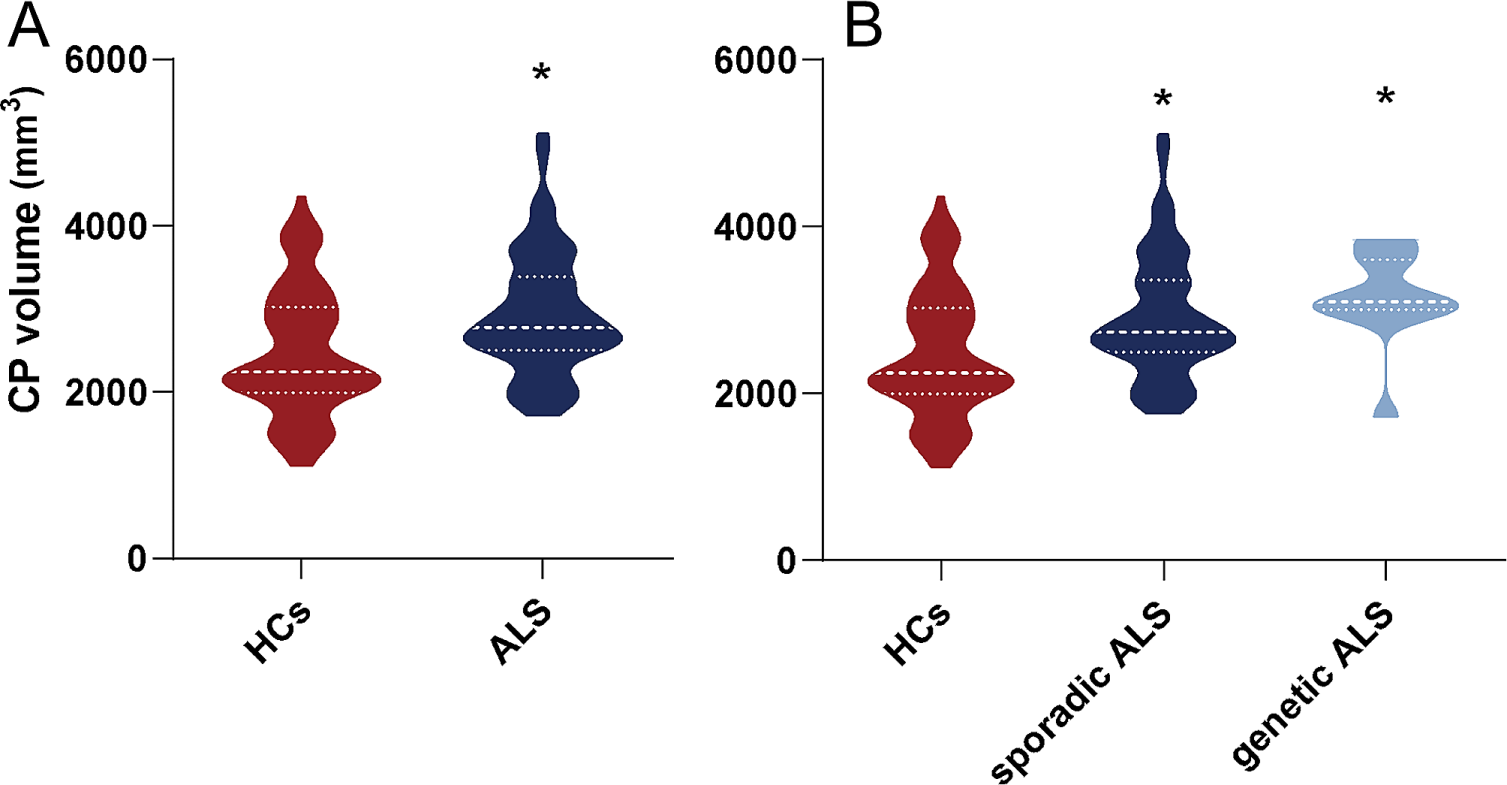
The CP volume in ALS patients and HCs. (**A**) Compared with HCs, ALS patients had a significantly higher CP volume after FWE correction (*p* < 0.05). (**B**) Compared with HCs, CP volume was significantly increased in genetic ALS patients and sporadic ALS patients after FWE correction (*p* < 0.05). Volume (mm3). Abbreviations: ALS = amyotrophic lateral sclerosis; CP = choroid plexus; HC = healthy control

In the present study, compared with HCs, ALS patients had a significantly higher CP volume (*p* < 0.001). The CP volumes of ALS patients and HCs are presented in Fig. 3A.

Moreover, compared with HCs, CP volume was significantly increased in genetic ALS patients and sporadic ALS patients after FWE correction (*p* = 0.006 and *p* < 0.001, respectively), while there were no significant differences between the two ALS groups. The CP volumes of the two ALS groups and HCs are presented in Fig. 3B.

### The correlation of CP volume with clinical disability and Qalb in ALS patients

**Fig. 4:**
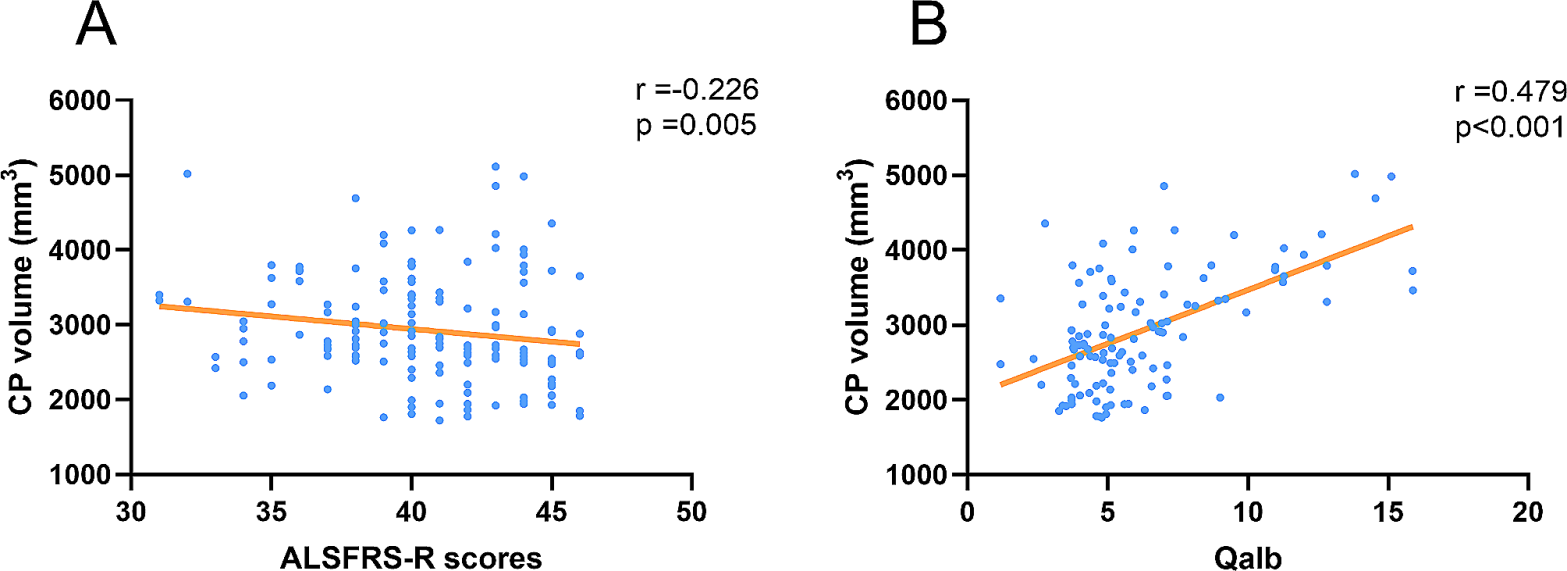
The correlation of CP volume with clinical disability and BCSFB permeability in ALS patients. (**A**) Association of CP volume and ALSFRS-r scores in ALS patients. (**B**) Association of CP volume and Qalb value in ALS patients. Volume (mm3). Abbreviations: ALS = amyotrophic lateral sclerosis; CP = choroid plexus

Finally, partial correlation analyses showed that the CP volume was significantly correlated with the ALSFRS-r scores (*r* = -0.226; *p* = 0.005) and Qalb (*r* = 0.479; *p* < 0.001). There were no correlations between CP volume and other metrics in this study. The relationship of CP volume with clinical disability and BCSFB permeability are presented in Fig. 4.

## Discussion

To our knowledge, we have conducted the first study, based on structural MRI, demonstrating in vivo CP enlargement in a large newly diagnosed cohort of ALS patients. Moreover, we found that CP enlargement was correlated with clinical disability and BCSFB permeability in ALS patients. We suggest that CP volume is noninvasive and easy-to-implement marker for screening BCSFB dysfunction in ALS patients. Thus, our findings may have an important role in advancing our current understanding of the pathophysiological process underlying ALS and suggest that the CP is likely an important target in future research.

In the present study, using Gaussian mixture models combined with a further manual correction approach, we found that, compared with HCs, the CP volume was significantly enlarged in ALS patients [18]. We further found that both sporadic ALS patients and genetic ALS patients had a significantly larger CP volume than HCs. Moreover, although genetic ALS patients had a larger mean CP volume than sporadic ALS patients, the difference did not reach statistical significance which may be due to the small sample size of the genetic group. To the best of our knowledge, no previous study to date has systematically assessed CP volume abnormalities in vivo in ALS patients, particularly in genetic ALS patients [20–22]. Importantly, animal and postmortem studies strongly support our findings [20–22]. For example, Kunis et al. found that CP activation is impaired in an ALS SOD1 G93A mouse model [20]. Recently, in a postmortem study, Saul et al. reported that widespread CP disruptions could be detected in 11 sporadic ALS patients and 3 ALS patients associated with GGGGCC repeat expansion in C9orf72 [21]. Thus, these findings may provide complementary evidence, and suggest that in vivo CP enlargement is likely an important and common feature of ALS patients [20–21].

Moreover, as the mean disease duration of this newly diagnosed cohort was 13.6 months, we further suggest that CP enlargement is likely an early feature of ALS patients [34, 35]. To date, the diagnostic delay of ALS is commonly 9.1–27 months, and patients may thus frequently miss the opportunity to benefit from early neuroprotective intervention and participate in clinical trials [34]. To facilitate early diagnosis, the “Gold Coast” criteria were recently presented, and the diagnostic levels (definite, probable and possible ALS) were abandoned in the new criteria [36]. However, the diagnosis of ALS is still largely based on its clinical presentation at present [36]. Due to its noninvasive and easy-to-implement nature, we suggest that early measurement of CP volume may be a potential approach for improving the diagnostic delay in ALS, for example, CP volume may be considered as a potential variable in a diagnostic model in the future [37]. Moreover, because the onset of ALS seems to involve a multistep process with a long preclinical stage and widespread BCSFB dysfunction that can be detected even in the presymptomatic-stage of ALS, we suggest that, similar to other neurodegenerative diseases, CP enlargement may also emerge in earlier stages in ALS patients [2–5, 23, 24]. Thus, future studies should be conducted to further examine whether CP enlargement can be detected in the earlier stages of ALS patients, for example, in ALS patients at King’s stage 1 or even in presymptomatic-stage patients with ALS, and the diagnostic performance of the CP volume in distinguishing between ALS and ALS-mimic diseases [1, 5, 34, 38].

Another key finding of the present study was that CP volume was significantly correlated with ALSFRS-r scores and Qalb value in ALS patients. In line with our findings, CP enlargement has been already reported to be associated with clinical disability in other neurodegenerative conditions, including AD, PD, FTD and MS [13–18]. However, few studies have been conducted to determine the correlation between CP volume and BCSFB permeability in neurodegenerative diseases to date [13–18]. To our knowledge, only one previous study has focused on this topic [13]. Recently, using dynamic contrast-enhanced imaging and structural MRI, Choi et al. found that CP volume is associated with BCSFB permeability in the AD spectrum [13]. Our findings suggest that CP volume is a potential marker for monitoring BCSFB dysfunction in ALS patients. Due to its noninvasive and easy-to-implement nature, further studies should be conducted to verify the correlation in ALS and other neurodegenerative diseases.

Importantly, similar to other neurodegenerative diseases, we suggest that the impact of CP abnormalities on the neurodegenerative process of ALS may largely be mediated by the accumulation of neurotoxic proteins, such as cytokines and reactive oxygen species, in the CSF due to increased blood leakage and/or reduced clearance [13–22, 39]. CP is crucial for maintaining BCSFB function in the CNS [10–12]. Thus, impaired CP barrier permeability may lead to the leakage of blood-borne toxins from the periphery to the CSF [8–12, 39]. In particular, impaired BCSFB integrity was supported by elevated CSF to serum ratios of albumin in ALS patients [40]. Moreover, as the CP is an important part of the glymphatic system, CP abnormalities may partly through impair waste clearance further accelerating neurodegeneration in ALS patients [10–12]. However, future studies still need to further confirm our viewpoints.

Moreover, as our findings and those of others, we suggest that CP abnormalities may be a common feature in neurodegenerative diseases [13–18]. To date, similar to other neurodegenerative diseases, the drivers of CP abnormalities in ALS remain largely unclear [13–18]. Recently, Steinruecke et al. proposed that mitochondrial dysfunction, astrocyte abnormalities and inflammation may contribute to BCNSB abnormalities in ALS [23]. As the CP is an important part of the BCNSB system, we suggest that these factors may also be involved in CP abnormalities in ALS [23, 24]. In particular, in line with previous postmortem findings, we found that CP abnormalities can be detected in 12 genetic ALS patients, further supporting this hypothesis [21, 36–40]. Moreover, as the CP enlargement has also been observed in other neurodegenerative diseases, enlargement of the CP may be a secondary consequence of larger ventricles in atrophic brains [13–15]. However, it is worth noting that very little is currently known about the causal relationships of CP abnormalities with these mechanisms underlying ALS; these topics should be further clarified in the future, which may be important for advancing our further understanding of the pathogenesis of ALS and other neurodegenerative diseases [20–22].

Overall, as we mention above, to date, very few studies have focused on CP abnormalities in ALS, and our findings may provide important information on this topic for future studies [20–22]. However, inevitably, the present study had several limitations. First, although our sample size was large and the clinical and epidemiological features of our ALS patients were consistent with those of a recent national population-based study, which may thus strengthen the validity and credibility of our results, the present study was a single centre study [27]. Thus, we suggest that further population-based or multicenter studies should be conducted to confirm our findings. Second, in this retrospective analysis, we could not analyze the causal relationship of CP abnormalities with clinical disability and brain damage in ALS. Thus, we suggest that longitudinal studies should be conducted in the future. Third, because few Chinese ALS patients carried a known ALS mutation, only 12 ALS mutation gene carriers were identified in our sample [28-30]. Thus, unfortunately, we could not determine the contribution of special mutations to the CP abnormalities in ALS, such as, SOD1, FUS and, in particular, C9orf72 [1, 29, 31–33]. Our findings need to be confirmed in a large sample of these ALS mutation gene carriers in the future. Finally, the racial composition of our cohorts was Chinese population; further studies should be conducted to identify whether CP abnormalities may emerge in Caucasian and other races of patients with ALS.

## Conclusions

Our study provides evidence that in vivo early CP enlargement is a common feature of ALS patients and continues to suggest an important pathogenetic role for CP abnormalities in ALS. Moreover, CP volume is likely a noninvasive and easy-to-implement approach for screening BCSFB dysfunction in ALS patients. Our findings may have an important role in advancing our current understanding of the pathophysiological process underlying ALS and the common pathways underlying neurodegenerative diseases. Future studies are needed to confirm our findings and further explore the mechanisms of CP enlargement in patients with ALS and other neurodegenerative diseases.

### Electronic supplementary material

Below is the link to the electronic supplementary material.

Supplementary Material 1

Supplementary Material 2

## Data Availability

The anonymized data presented in this article are available at the request of a qualified investigator, after review by the corresponding author. Final approval will be granted by the Research Ethics Committee of the School of Medicine, Shandong University.

## Declarations

### Statistical analysis

Statistical analysis was conducted by Dr. Xiaohan Sun, PhD, Department of Neurology, Qilu Hospital of Shandong University, Jinan, China

## Abbreviations

ALS: Amyotrophic lateral sclerosis
BCSFB: Blood-CSF barrier
CP: Choroid plexus
FUS: Fused in sarcoma
SOD1: Superoxide dismutase 1
