# Supplementary material for: Choroid plexus enlargement in amyotrophic lateral sclerosis patients and its correlation with clinical disability and blood-CSF barrier permeability
Source: Fluids Barriers CNS. 2024 Apr 17;21:36. doi: 10.1186/s12987-024-00536-6 (PMC11025206; doi:10.1186/s12987-024-00536-6)
Supplement: Supplementary file 1 — Supplementary Material 1 [file 12987_2024_536_MOESM1_ESM.docx]

**Supplemental material**

**Genetic testing**

In the present study, all ALS patients underwent genetic testing. Similar to our previous study, 32 ALS genes were also screened using whole exome sequencing (WES) in ALS patients, including the genes SOD1, ALS2, SETX, FUS, VAPB, TARDBP, OPTN, VCP, UBQLN2, SIGMAR1, FIG4, CHMP2B, PFN1, NEFH, PRPH, TFG, TAF15, GRN, CHCHD10, TUBA4A, TBK1, NEK1, GLE1, MATR3, CCNF, ANXA11, HNRNPA1, SQSTM1, ERBB4, TIA1, SPG11 and KIF5A, according to the ALS online database and previous reports. The procedures have been documented in detail in our previous study.^1^ Briefly, the criteria used to detect causative mutations were based on the recommendations of the American College of Medical Genetics. Variant frequencies were initially determined using gnomeAD and the Exome Aggregation Consortium to remove the common single nucleotide polymorphisms. Only non-synonymous, splicing and frameshift variants with a minor allele frequency (MAF) of less than 0.5% across all population databases were selected for further analysis. Sanger sequencing was performed to further validate the mutations identified by WES. C9orf72 repeat expansions were also screened using standard repeat-primed PCR.^1^

**References**

1 Sun X, Zhao X, Liu Q, et al. Study on sleep-wake disorders in patients with genetic and non-genetic amyotrophic lateral sclerosis. J Neurol Neurosurg Psychiatry. 2020 Oct 21:jnnp-2020-324544.
