# Supplementary material for: Choroid plexus enlargement in amyotrophic lateral sclerosis patients and its correlation with clinical disability and blood-CSF barrier permeability
Source: Fluids Barriers CNS. 2024 Apr 17;21:36. doi: 10.1186/s12987-024-00536-6 (PMC11025206; doi:10.1186/s12987-024-00536-6)
Supplement: Supplementary file 2 — Supplementary Material 2 [file 12987_2024_536_MOESM2_ESM.docx]

Supplementary Table 1. Genetic data of patients carrying ALS gene mutations

| No. | Mutant gene | Nucleotide change | Amino acid change |
| --- | --- | --- | --- |
| Patient 1 | SOD1 | c.251A>G | p.A84G |
| Patient 2 | SOD1 | c.140A>G | p.H47A |
| Patient 3 | SOD1 | c.143T>C | p.V48A |
| Patient 4 | SOD1 | c.217G>C | p.G73R |
| Patient 5 | SOD1 | c.185G>C | p.G62A |
| Patient 6 | SOD1 | c.335G>A | p.C112T |
| Patient 7 | FUS | c.1561C>T | p.R521C |
| Patient 8 | FUS | c.703A>G | p.S235G |
| Patient 9 | FUS | c.1561C>T | p.R521C |
| Patient 10 | FUS | c.1574C>T | p.P525L |
| Patient 11 | TBK1 | c.34T>G | p.S12A |
| Patient 12 | VCP | c.463C>T | p.R155C |

Abbreviations: ALS = amyotrophic lateral sclerosis.
